# Guild-based approach for mitigating information loss and distortion issues in microbiome analysis

**DOI:** 10.1172/JCI185395

**Published:** 2024-09-03

**Authors:** Liping Zhao, Guojun Wu, Naisi Zhao

**Affiliations:** 1 Department of Biochemistry and Microbiology, School of Environmental and Biological Sciences and Center for Microbiome, Nutrition, and Health, New Jersey Institute for Food, Nutrition, and Health, Rutgers, The State University of New Jersey, New Brunswick, New Jersey, USA.; 2 State Key Laboratory of Microbial Metabolism and Ministry of Education Key Laboratory of Systems Biomedicine, School of Life Sciences and Biotechnology, Shanghai Jiao Tong University, Shanghai, China.; 3 Department of Public Health and Community Medicine, School of Medicine, Tufts University, Boston, Massachusetts, USA.

## Introduction

Microbiome research holds the promise of elucidating new mechanisms of disease development and developing innovative approaches for disease prevention and treatment. However, a substantial challenge that stymies this potential is the often overlooked issue of information loss and distortion during data analysis. Such analytical shortcomings are a primary contributor to inconsistencies between studies. For example, conflicting findings often emerge, such as divergent taxa linked to the same diseases in separate studies (1, 2). Likewise, the same taxon may have contrasting associations with identical diseases across different investigations (2, 3). This issue is exemplified by the phylum Firmicutes, which has been linked to both an increase and a decrease in prevalence of type 2 diabetes across different studies (4, 5). Similar inconsistencies are found at the genus level; conflicting reports exist regarding the role of Collinsella in autism spectrum disorder. While studies by Strati et al. (6) and Chamtouri et al. (7) found a positive association (detrimental effects) between Collinsella and autism spectrum disorder, other researchers showed a reduction of Collinsella (8, 9). Such inconsistencies are common in microbiome studies on various diseases (2).

The root of these inconsistencies often lies in the insufficient recognition of the profound genetic and functional diversity present at the strain level within a single bacterial species. The average nucleotide identity within a bacterial species can vary by 4% to 5% (10), a striking contrast when compared with the approximate 1% genomic difference between humans and chimpanzees (11). This heterogeneity within a bacterial species demands our attention, for it holds the key to comprehending the delicate balance within microbiomes. Despite advances in technology that allow for analyses at finer granularities, such as amplicon sequence variant (ASV) (12) and metagenome-assembled genome (MAG) (13), conventional data analysis methodologies in microbiome research often fail to account for this strain-level variation. This oversight leads to a cascade of information loss and distortion, ultimately impeding our comprehension of the intricate connections between the microbiome and human health.

**Figure 1:**
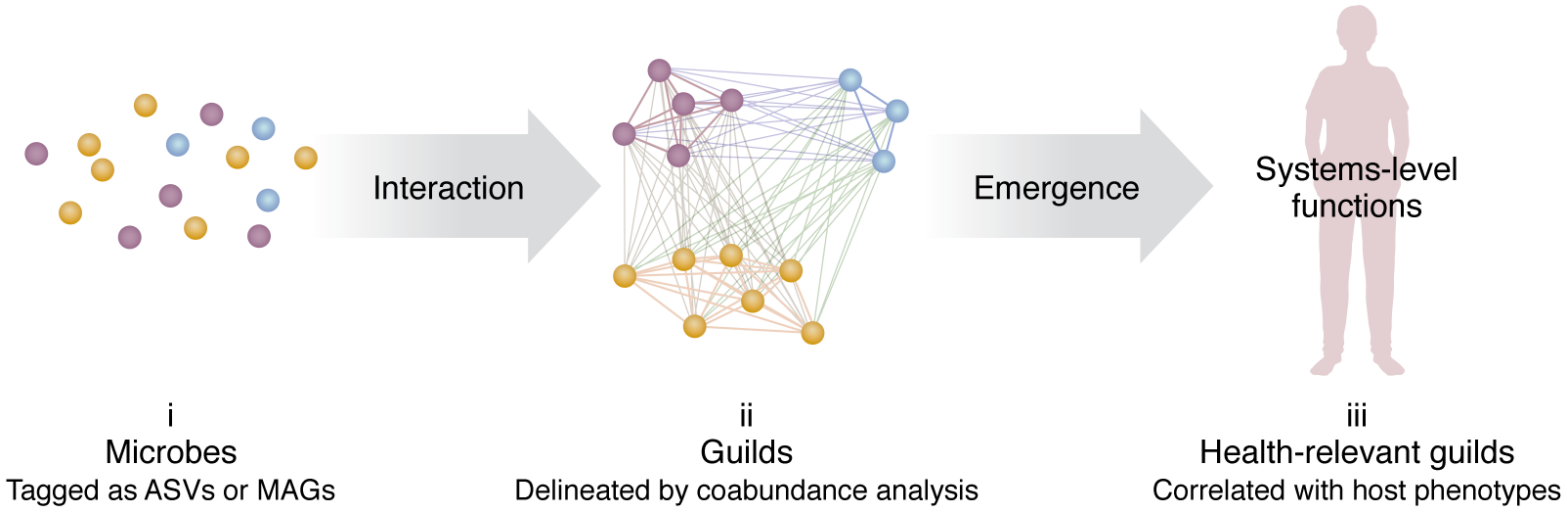
Guild-based analysis of microbiome datasets. A guild consists of microbes with diverse taxonomic backgrounds, but thriving or declining together, showing coabundance behavior. (i) Initial tagging and identification of individual microbial entities using ASVs or MAGs, each assigned a UUID for precise tracking. (ii) Analysis of interactions among microbial entities to identify patterns of coexistence and influence, revealing the foundational relationships within the microbiome. Clustering of microbes into guilds based on coabundance analysis, where members share ecological niches and exhibit similar abundance patterns across different conditions or samples. (iii) Emergence of complex microbial behaviors and functions from the interactions within and between guilds, highlighting the collective capabilities of the microbiome. Integration of guild activities into broader systems-level functions that affect host physiology and health, encapsulating the holistic effect of microbial interactions.

In this Viewpoint article, we will dissect the limitations that hinder our strain-level understanding, delve into tools for evaluating information loss and distortion, and advocate for a genome-centric and guild-based approach to mitigating these issues (Figure 1). Such a paradigm shift is not only about refining technical approaches, but is also about adopting a new perspective that can enhance the integrity and applicability of microbiome research.

## Evaluating information loss and distortion

Microbiome analysis generates extensive datasets composed of unique sequences, such as ASVs or MAGs, each representing unique types of microbes. These datasets encapsulate a wealth of information about microbial diversity and functionality within microbiome samples. Nevertheless, the high dimensionality and sparsity of these datasets present significant challenges. With variables outnumbering samples, a phenomenon known as the “curse of dimensionality” emerges, complicating the identification of authentic health-related microbial signatures (14). Thus, reducing the dimensionality and sparsity of the original microbiome datasets, collectively called data reduction, is imperative for microbiome analysis. However, information loss and distortion can occur in current data reduction practices in mainstream microbiome analysis.

### Information loss.

Information loss on novel or understudied microbes and their functions can occur in database-dependent analysis of microbiome datasets. The primary step in conventional microbiome data analysis involves taxonomic assignment and functional annotation, heavily relying on reference databases such as SILVA (15) (https://www.arb-silva.de/) or KEGG (16) (https://www.genome.jp/kegg/). When unclassified or unannotated sequences are excluded from downstream analysis, information on the diversity and function of novel or understudied microbes they represent will be ignored in any further analysis. In practice, it’s common for 10%–40% of ASVs to remain unclassifiable at the genus level, and up to 50% of genes may lack functional annotations (17). This exclusion can result in a substantial portion of data being disregarded, thus potentially skewing the representation of microbial communities and functions.

### Information distortion.

Information distortion, on the other hand, happens when the process of reducing dataset complexity introduces biases. For instance, lumping ASVs by genus or genes by pathways (14) can conceal the nuances of strain-level variation. Strains within the same taxon may exhibit different or opposing correlations with the same disease or intervention. Similarly, the same critical pathway gene, such as the *but* gene for butyrate production, may be harbored by two competing bacterial strains, masking the true abundance change of this gene in microbiome datasets (18). Failure to account for these strain-level variations during dimensionality reduction can lead to information distortion, resulting in inconsistencies across microbiome studies and hindering the establishment of clear associations between microbiome features and diseases.

### A model for evaluating information loss and distortion.

To address these challenges, we propose employing β diversity matrices of all ASVs or MAGs as a benchmark for the entire information content of the original datasets. We then advocate for the combined use of Procrustes analysis (19) and the Mantel test (20) to evaluate information loss and distortion, which may happen after each attempt at data reduction. These methods can compare and assess the similarity or dissimilarity between multivariate datasets. A close match between the β diversity matrices before and after data reduction indicates successful preservation of original dataset characteristics. At the same time, a significant difference may signal a loss or misrepresentation of information. For example, a new β diversity matrix based on genus-level variables should be created when analyzing ASV datasets at the genus level. This new matrix needs to be compared with the original one at the ASV level using Procrustes analysis and the Mantel test. If the matrices show congruence, it suggests minimal information loss and distortion, indicating that the reduced dataset accurately represents the original. Conversely, the pronounced disparity between the matrices may indicate potential information misrepresentation. In addition, when comparing different data reduction methods, the combined use of Procrustes analysis and the Mantel test can help determine which method better preserves information from the original datasets.

These methods ensure that dimensionality reduction maintains data integrity. By preserving the essence of information in the original datasets while reducing complexity, researchers can generate more reproducible and consistent results for microbiome biomarker discovery.

## Mitigating information loss and distortion

We advocate for a guild-based analytical strategy to confront the pervasive issues of information loss and distortion in microbiome analysis (17). This innovative approach transcends the confines of traditional methods, offering a precise and ecologically sound representation of microbial communities. The guild-based approach is supported by three key pillars.

### Genome-specific analysis.

Microbial cells and viral particles are the fundamental units of change at the core of the gut ecosystem. A genome-specific approach leverages genomic data as molecular tags to track and catalog the entire microbial constituency. Advancements in sequencing technologies are bringing us closer to a future where comprehensive genomic mapping of microbiomes becomes feasible and cost-effective. Until then, ASVs or MAGs with a 1% average nucleotide identity (ANI) difference are proxies for such detail, allowing near strain-level resolution.

### Database-independent inclusivity.

To curtail information loss inherent in database-dependent analyses, we can implement a system of universal unique identifiers (UUIDs) for each MAG or ASV, streamlining tracking across samples and studies. The generation of UUIDs is solely based on the sequence identity between MAGs or ASVs. New UUIDs will be assigned if the novel MAGs and ASVs are not found in existing studies. With such a UUID system in place, taxonomic assignment or functional annotation will not be the primary step in microbiome analysis. Thus, novel microbes will not be excluded from downstream analysis. This reference-free approach ensures that our analysis remains unbiased toward known species, enabling the discovery of previously unidentified or understudied microbes. By embracing the unknown, we achieve a more inclusive and comprehensive view of the microbiome, reducing information loss related to database limitations.

### Interaction-focused aggregation.

In the intricate web of the gut ecosystem, microbes do not exist in isolation, but rather form synergistic collectives known as guilds. Members in the same guild cooperate, thrive, or decline together, showing coabundance behavior. Different guilds may cooperate or compete to form the whole ecosystem network. We introduce guild-level categorization as the primary method for dimensionality reduction in microbiome analysis (17), focusing on functional groupings within ecosystems, which consider the complex interactions between microbes. Members in these guilds can be clustered together based on their coabundance behavior, irrespective of their taxonomic background. This perspective considers the ecological interactions and cooperative relationships among microbes, providing insights into how groups work together to influence microbiome stability and function. This approach ensures that valuable functional insights are not obscured by taxonomic lumping, minimizing the information distortion.

The guild-based approach ushers in a more objective, holistic, and functionally oriented understanding of microbial communities and their impact on human health. This framework has revealed bacterial guilds’ potential role in disease phenotype development, such as obesity in Prader-Willi syndrome (21), and uncovered microbial guilds alleviating type 2 diabetes when fostered by dietary fibers (18). Our recent study further demonstrates the power of the guild-based analytical approach, structured around the three methodological pillars: genome specificity, database independence, and interaction-focused aggregation. By focusing on stably correlated genomes, we identified a core microbiome characterized by two competing guilds, one beneficial, the other detrimental. This core structure persisted despite the diverse confounding factors inherent in microbiome datasets spanning various studies with wide variations of interventions, diseases, geographic locations, ethnicities, and sequencing protocols. This finding underscores the robustness of the guild-based approach in capturing fundamental microbiome patterns that are crucial for understanding human health (22). By implementing this approach, we can effectively mitigate information loss and distortion, thereby enhancing the robustness and reproducibility of microbiome research and improving the validity of our findings across studies.

## Conclusion

As microbiome research advances, the imperative to overcome information loss and distortion becomes increasingly critical. The genome-centric and guild-based methodologies represent our commitment to this cause. We aspire to mitigate these challenges by adopting genome-centric and guild-based analysis. In this quest for precision and comprehensiveness, we extend an invitation to the global research community. We call upon the global research community to join in refining these approaches, thus fortifying the integrity of microbiome research and catalyzing breakthroughs in disease prevention, diagnosis, and treatment, with far-reaching implications spanning science, medicine, and beyond.

## Version 1 09/03/2024

Electronic publication
